# Effect of partial restorative treatment on stress distributions in non-carious cervical lesions: a three-dimensional finite element analysis

**DOI:** 10.1186/s12903-022-02647-8

**Published:** 2022-12-15

**Authors:** Xin Luo, Qiguo Rong, Qingxian Luan, Xiaoqian Yu

**Affiliations:** 1 Department of Periodontology, Peking University School and Hospital of Stomatology, No. 22, Zhongguancun South Avenue, Haidian District, Beijing, 100081 China; 2 Department of Mechanics and Engineering Science, College of Engineering, Peking University, Beijing, China

**Keywords:** Partial restorations, Non-carious cervical lesions (NCCLs), Finite element analysis, Restoration bond failures, Composite resin (CR), Glass-ionomer cement (GIC), Mineral trioxide aggregate (MTA)

## Abstract

**Background:**

Partial restoration combined with periodontal root coverage surgery can be applied to the treatment of non-carious cervical lesions (NCCLs) accompanied with gingival recessions in clinical practice. However, the feasibility of NCCL partial restorative treatment from a biomechanical perspective remains unclear. This study aimed to investigate the effect of partial restorations on stress distributions in the NCCLs of mandibular first premolars via three-dimensional finite element analysis.

**Methods:**

Three-dimensional finite element models of buccal wedge-shaped NCCLs in various locations of a defected zenith (0 mm, 1 mm, and 2 mm) were constructed and divided into three groups (A, B, and C). Three partially restored NCCL models with different locations of the lower restoration border (1 mm, 1.5 mm, and 2 mm), and one completely restored NCCL model were further constructed for each group. The following restorative materials were used in all restoration models: composite resin (CR), glass-ionomer cement (GIC), and mineral trioxide aggregate (MTA). The first principal stress distributions under buccal oblique loads of 100 N were analyzed. Restoration bond failures were also evaluated based on stress distributions at dentin-restoration interfaces.

**Results:**

When the partial restoration fully covered the defected zenith, the first principal stress around the zenith decreased and the maximum tensile stress was concentrated at the lower restoration border. When the partial restoration did not cover the defected zenith, the first principal stress distribution patterns were similar to those in unrestored models, with the maximum tensile stress remaining concentrated at the zenith. As the elastic modulus of the restorative material was altered, the stress distributions at the interface were not obviously changed. Restoration bond failures were not observed in CR, but occurred in GIC and MTA in most models.

**Conclusions:**

Partial restorations that fully covered defected zeniths improved the stress distributions in NCCLs, while the stress distributions were unchanged or worsened under other circumstances. CR was the optimal material for partial restorations compared to GIC and MTA.

## Background

Non-carious cervical lesions (NCCLs) are described as the irreversible loss of hard dental tissues at the cementoenamel junction (CEJ) unrelated to dental caries; they have a mean prevalence of 46.7% [1]. They can be treated by complete restorations to fully recover the lost dental structures and relieve the stress concentration around defects. However, almost half of NCCLs are associated with gingival recessions, leading to combined defects (CDs) [2]. In this scenario, conventional restorations alone cannot address the esthetic problems attributable to gingival recessions, despite resolving the hard tissue defect [3].

In order to address the esthetic issues, periodontal root coverage surgery should be considered [4]. Combining restorative treatment with periodontal root coverage surgery is applicable to CDs, resolving both defects [5, 6]. The detailed procedures of the combined restorative-surgery approach have been documented in previous clinical studies [7, 8]. In brief, the maximum root coverage level (MRC) achieved by periodontal surgery is estimated [3], then the coronal portion of the NCCL is partially restored, with the lower restoration border placed up to 1 mm apically to the MRC. Periodontal root coverage surgery is performed thereafter to cover the remaining exposed root surfaces.

Although the clinical outcomes of the combined restorative-surgery approach have been verified by numerous randomized controlled clinical trials [8–10], the influence of partial restorations on stress distributions in NCCLs remains unclear. It is necessary to analyze the stress distributions in partially restored NCCLs to comprehensively evaluate the feasibility of partial restorations. Furthermore, little information is presently available on the debonding risk of partial NCCL restorations, which is affected by the stress distributions at dentin-restoration interfaces. Therefore, analyzing the stress distributions in partially restored NCCLs will provide essential information to assist in the balancing of esthetics and biomechanics during the decision-making process of CD treatment in clinical practice.

Using a three-dimensional finite element (FE) analysis, this study aimed to investigate the effect of partial restorations on stress distributions in NCCLs. In addition to conventionally used restorative filling materials, MTA was also investigated due to its increased application in endodontic repair including internal root resorption cavities and iatrogenic root perforations [11, 12].

## Material and methods

### Mandibular first premolar model construction

A microcomputed tomography image of a mandibular first premolar from the database of Digital Dentistry Center in Peking University School of Stomatology, Beijing, China, was obtained. The geometric data were transformed into Digital Imaging and Communications in Medicine format. An interactive medical image control system (Mimics 15.0; Materialise, Leuven, Belgium) was then used to separate this geometric data into three portions, comprising the enamel, dentin, and pulp, based on their different gray value thresholds. These three segmented portions were then imported into reverse engineering software (Geomagic Studio; Geomagic Inc, Morrisville, NC, USA) to generate a solid FE model. A cuboid was artificially generated around the root to simulate the mandibular alveolar bone, with the upper border ending 3.5 mm below the cementum-enamel junction (CEJ) and a 2 mm thickness of the cortical bone [13]. The periodontal ligament with a thickness of 0.3 mm was also modelled to connect the alveolar bone and the root. The root cementum was not generated due to the negligibly small dimensions and limited relevance to the research [14]. The reconstructed FE model of the intact mandibular first premolar was defined as model Sound (S).

### Unrestored and restored defect model construction

**Fig. 1:**
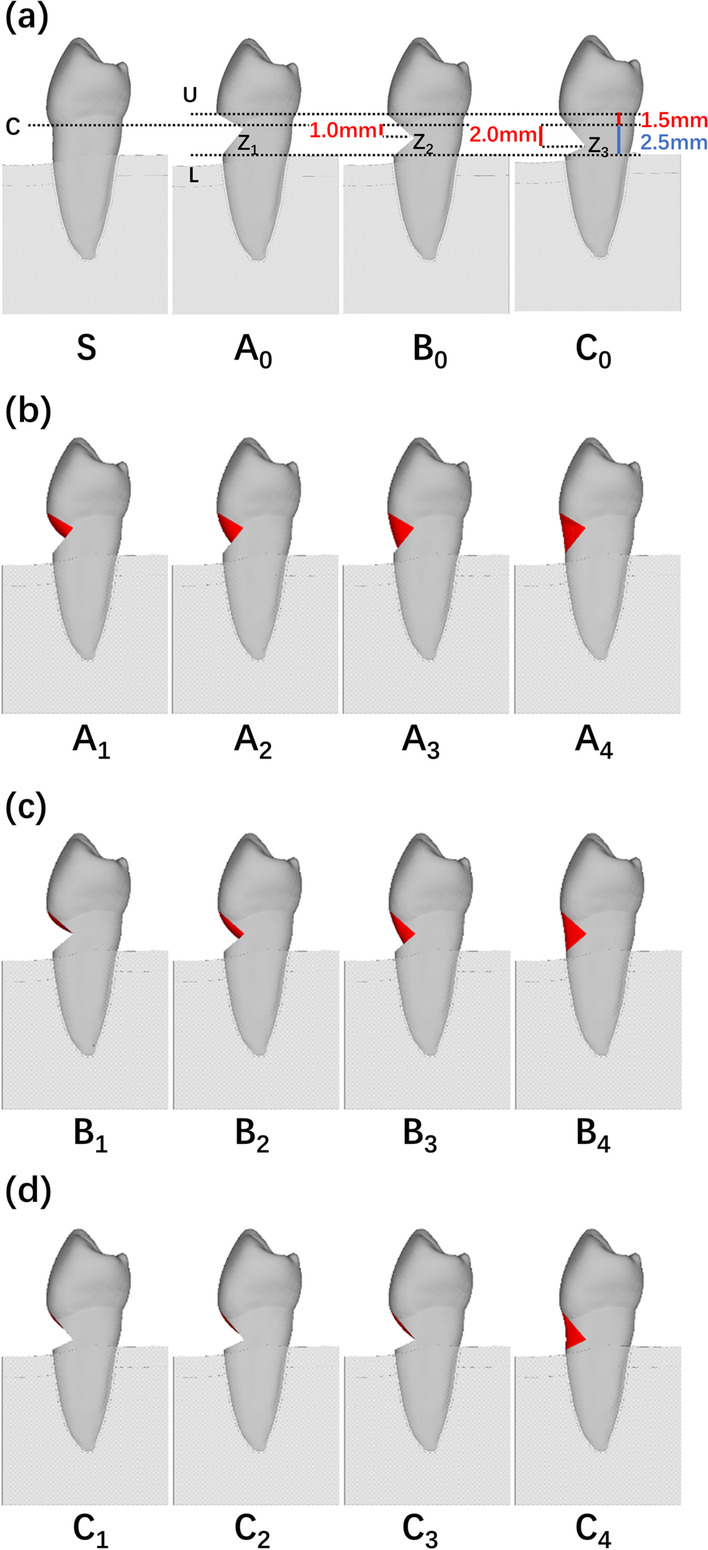
Schematic representation of the finite element models. **a** Detailed parameters of unrestored NCCLs (A_0_, B_0_, C_0_); **b** partially restored NCCLs (A_1_–A_3_) and a completely restored NCCL (A_4_); **c** partially restored NCCLs (B_1_–B_3_) and a completely restored NCCL (B_4_); **d** partially restored NCCLs (C_1_–C_3_) and a completely restored NCCL (C_4_)

Three buccal wedge-shaped NCCL models were designed based on model S (Fig. 1a). The buccal midpoint of the CEJ (point C) was chosen as the reference point during the construction of all models. The midpoint of the upper NCCL margin (point U) was located 1.5 mm coronally away from point C, while the midpoint of the lower NCCL margin (point L) was 2.5 mm apically away from point C in the *z* direction. The NCCL height, defined as the distance between point U and point L in the *z* direction, was fixed at 4 cm. The depth of the NCCL, defined as the distance between point C and the midpoint of the defected zenith (point Z) in the *y* direction, was fixed at 2 mm. Three unrestored NCCL models with varying locations of point Z were created as negative controls: model A_0_ with point Z_1_ located at the same level as point C, model B_0_ with point Z_2_ located 1 mm apically away from point C, and model C_0_ with point Z_3_ located 2 mm apically away from point C in the *z* direction. The mesial and distal borders of models A_0_, B_0_, and C_0_ were located within the buccal-proximal axes of the premolar.

Partially restored NCCL models were further created based on models A_0_, B_0_, and C_0_ (Fig. 1b–d). According to clinical procedures [7, 15], the lower restoration border should be placed up to 1 mm apically to the MRC and complete root coverage can rarely be fulfilled [16, 17]. Therefore, nine partially restored NCCL models with different locations of the lower restoration border were created: models A_1_, B_1_, and C_1_ with the midpoint of the lower border located 1 mm apically to point C, as the most ideal circumstance; models A_2_, B_2_, and C_2_ with the midpoint of the lower border located 1.5 mm apically to point C; and models A_3_, B_3_, and C_3_ with the midpoint of the lower border located 2 mm apically to point C in the *z* direction. The lower partial restoration borders were designed as symmetrical curved lines with an arc height of 0.25 mm. Completely restored NCCL models A_4_, B_4_, and C_4_ were also constructed as positive controls (Fig. 1b–d). The geometric properties, classifications, and numbers of elements and nodes of the above models are shown in Table 1.

**Table 1:** Geometric properties, classifications, and numbers of elements and nodes for each model

Defect	Defect sizes (mm)				Model	Restorative method	Vertical distances of C-lower restoration border (mm)	Elements	Nodes
	Vertical distances			Horizontal distances					
	C-U	C-L	C-Z	C-Z					
A	1.5	2.5	0	2	A_0_	Unrestored		241,899	535,327
					A_1_	Partial	1	241,899	536,435
					A_2_	Partial	1.5	241,614	534,272
					A_3_	Partial	2	240,282	532,595
					A_4_	Complete	2.5	239,946	531,749
B	1.5	2.5	1	2	B_0_	Unrestored		239,350	530,829
					B_1_	Partial	1	239,350	531,328
					B_2_	Partial	1.5	241,898	534,001
					B_3_	Partial	2	240,145	532,165
					B_4_	Complete	2.5	241,613	533,027
C	1.5	2.5	2	2	C_0_	Unrestored		239,267	530,709
					C_1_	Partial	1	239,267	530,992
					C_2_	Partial	1.5	239,764	531,940
					C_3_	Partial	2	240,228	531,855
					C_4_	Complete	2.5	245,353	536,797

The chosen restorative materials were composite resin (CR; Filter Z250 XT Nano Hybrid Universal Restorative, 3 M ESPE, St Paul, MN, USA), glass-ionomer cement (GIC; GC Fuji II, Tokyo, Japan), and mineral trioxide aggregate (MTA; ProRoot MTA, Dentsply, Tulsa, OK, USA). The adhesive layer of the CR adhesive was not modelled due to the thinness [18] and similar elastic modulus to that of dentin. GIC and MTA could self-adhere to dental tissues without the need of adhesives. All models were assumed to have perfect adhesions among the restorations and dental tissues.

### Material properties

All dental tissues and restorative materials were assumed to be homogeneous and isotropic with linear elasticity. The mechanical properties of the dental tissues and restorative materials based on previous studies [14, 19–37] are summarized in Table 2.

**Table 2:** Material properties

Material	Elastic modulus (MPa)	Poisson’s ratio	Micro-tensile bond strength (MPa)	Shear bond strength (MPa)	Ultimate tensile strength (MPa)
Enamel [19]	84.1 × 10^3^	0.30			
Dentin [19]	18.6 × 10^3^	0.30			
Pulp [20, 21]	2.07	0.45			
Periodontal ligament [20, 21]	68.9	0.45			
Cortical bone [22, 37]	13.7 × 10^3^	0.30			
Cancellous bone [22, 37]	1.37 × 10^3^	0.30			
Composite resin (Filter Z250 XT) [23, 24]	16.6 × 10^3^	0.24			45.06
Glass-ionomer cement (GC Fuji II) [14, 25–28]	10.8 × 10^3^	0.30	9.30	6.30	11.80
Mineral trioxide aggregate [29–33]	15.7 × 10^3^	0.23	12.00	2.51	7.21
Adhesives (Clearfil SE Bond) [34–36]			60.00	25.30	92.80

### Boundary constraints and loading conditions

**Fig. 2:**
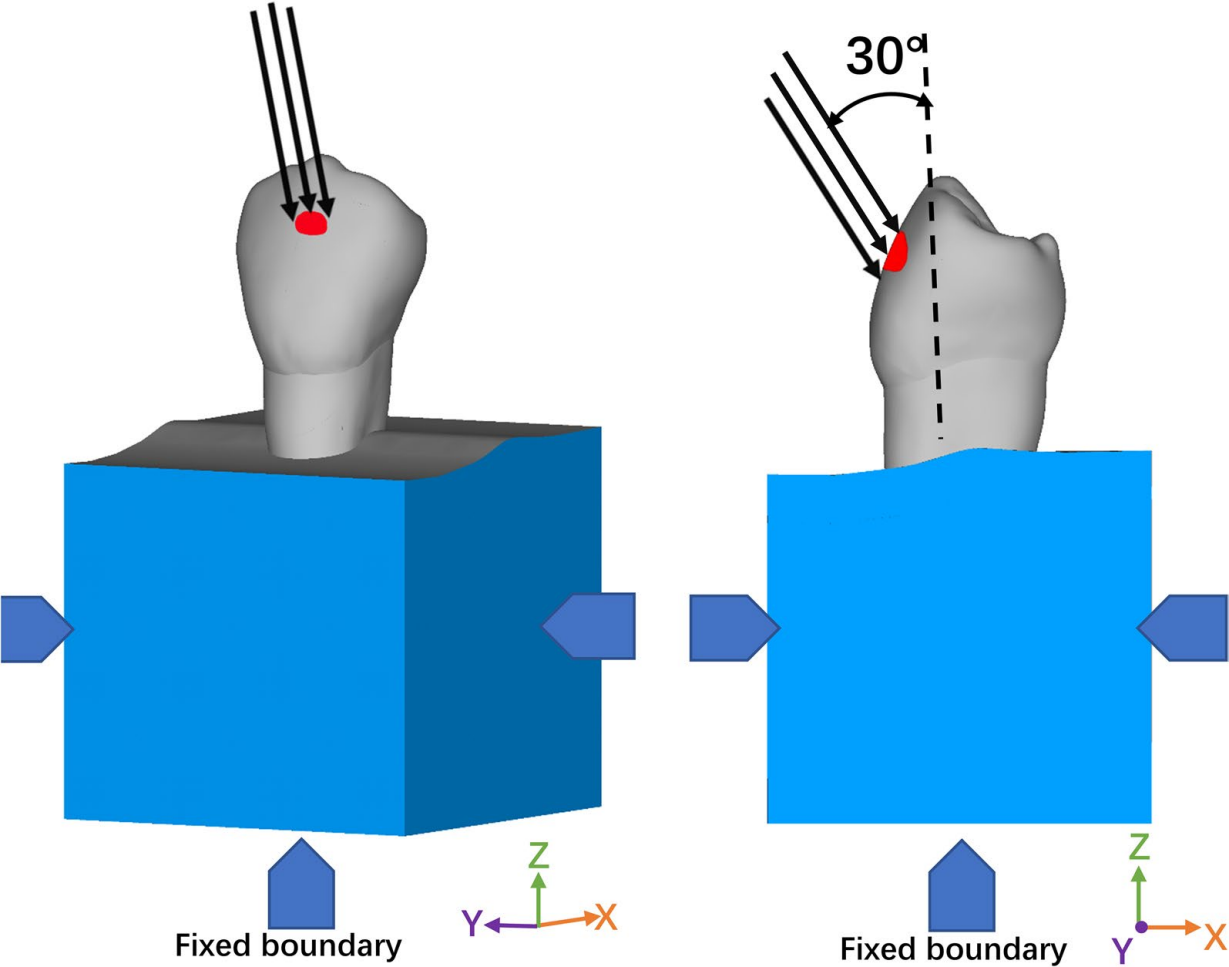
Schematic representation of the buccal oblique load (the black vector indicates the direction of the applied load. The blue areas indicate the fixed boundary conditions of the model at the lateral and basal surfaces.)

The lateral and basal surfaces of the alveolar bone were fixed in the x, y, and z directions. The contact types of bone, periodontal ligament, tooth, and repair materials were all bonded. Based on the results of previous studies [21, 23, 26, 28, 35, 36], a 100 N static oblique load, at 30° angle to the long axis of the premolar, was applied to the outer slope of the buccal cusp 1 mm away from the cusp tip to simulate lateral chewing movement (Fig. 2).

### Stress analysis

The above models were imported into a FE software (Ansys ver.16.0, ANSYS, Canonsburg, PA, USA) for stress analysis. Given that dental hard tissues have high compressive strength but low tensile strength [38], the first principal stress distribution was analyzed. Furthermore, in order to predict restoration debonding risks, the first principal stress and shear stress distribution at restoration-dentin interfaces were also analyzed. The maximum tensile stress (MTS) and the maximum shear stress (MSS) were further compared with the bond strength and ultimate tensile strength (UTS) of the corresponding materials.

## Results

### First principal stress distribution in dental structures

**Fig. 3:**
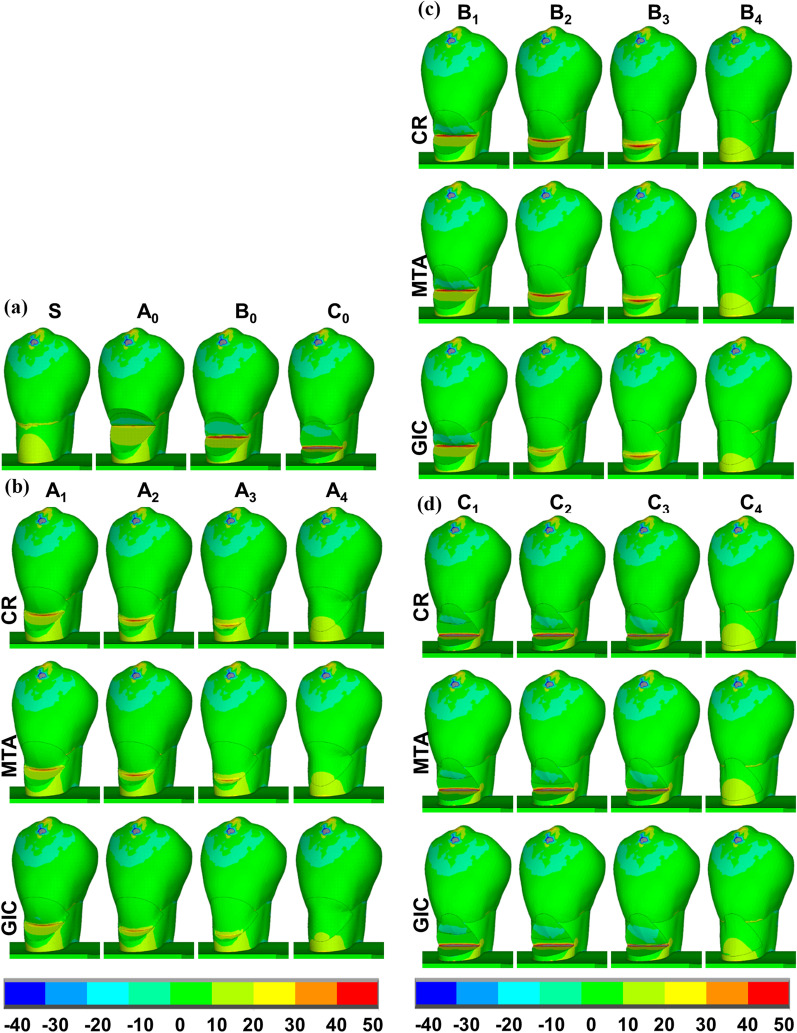
Specific regions analyzed for first principal stress (MPa) distributions in models using different restorative materials. **a** Natural tooth (S) and unrestored NCCLs (A_0_, B_0_, C_0_); **b** partially restored NCCLs (A_1_–A_3_)

**Fig. 4:**
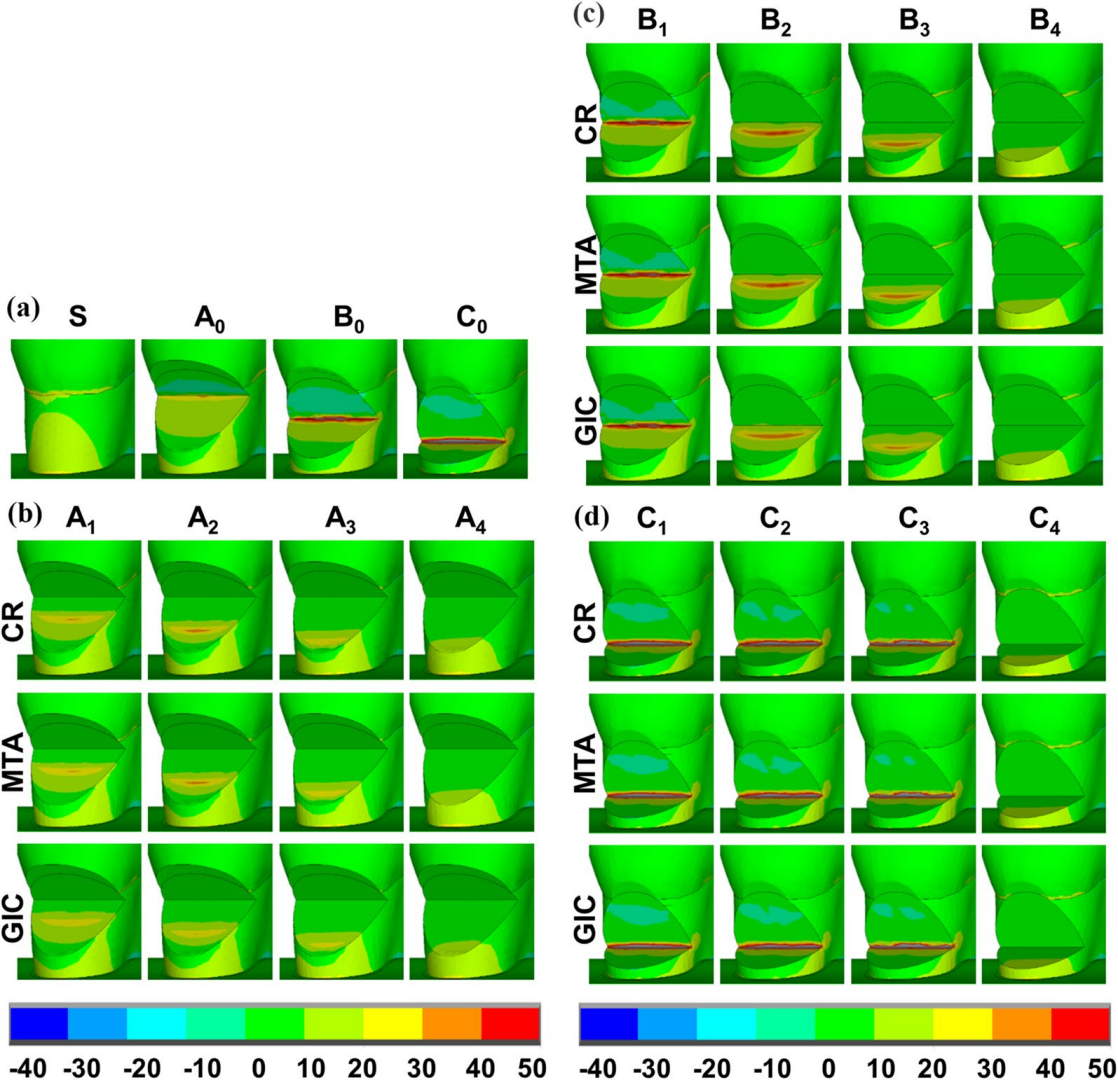
Close-up view of cervical regions for first principal stress (MPa) distributions in the models where restorations were placed on transparently to enable NCCL wall visualization. **a** Natural tooth (S) and unrestored NCCLs (A_0_, B_0_, C_0_); **b** partially restored NCCLs (A_1_–A_3_) and completely restored NCCLs (A_4_); **c** partially restored NCCLs (B_1_–B_3_) and completely restored NCCLs (B_4_); **d** partially restored NCCLs (C_1_–C_3_) and completely restored NCCLs (C_4_)

**Fig. 5:**
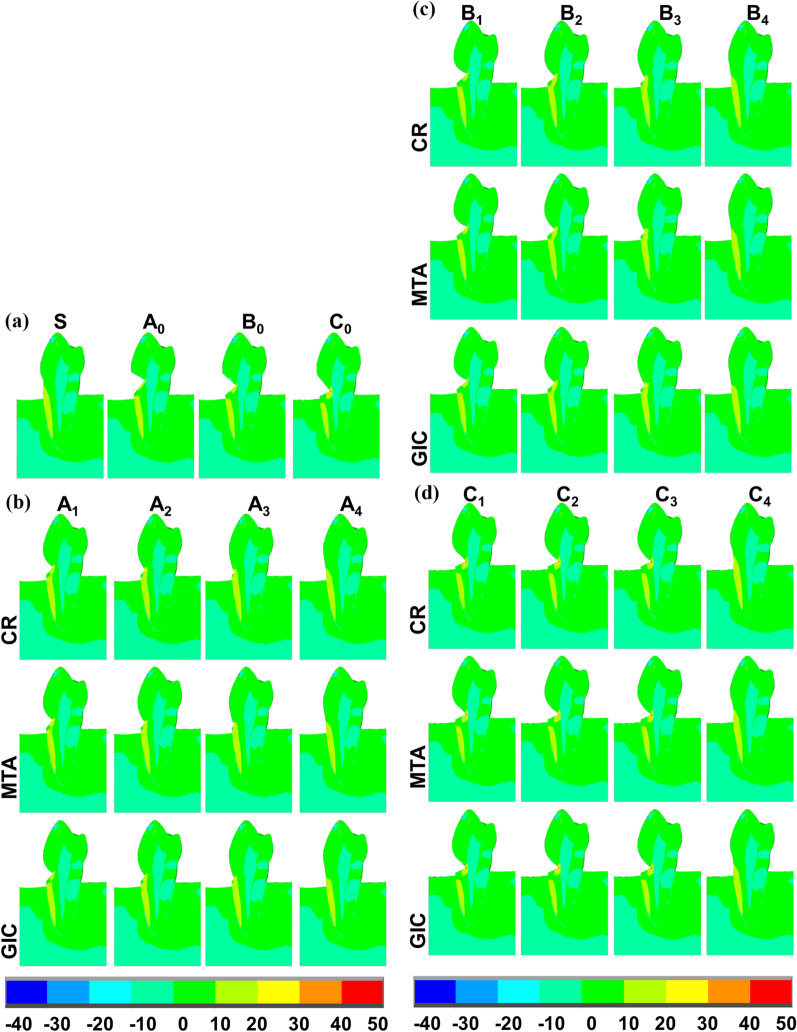
Sagittal sections of first principal stress (MPa) distributions in models using different restorative materials. **a** Natural tooth (S) and unrestored NCCLs (A_0_, B_0_, C_0_); **b** partially restored NCCLs (A_1_–A_3_) and completely restored NCCLs (A_4_); **c** partially restored NCCLs (B_1_–B_3_) and completely restored NCCLs (B_4_); **d** partially restored NCCLs (C_1_–C_3_) and completely restored

In the sound tooth, higher tensile stress was distributed at the cervix and the buccal root dentin with the MTS concentrated at the CEJ. In unrestored NCCLs, higher tensile stress was distributed on the lower defected wall with values decreasing from the zeniths to the lower defected border; the MTS was concentrated at the zeniths. In models A_4_, B_4_, and C_4_ restored by CR, the stress distribution patterns and MTS values were similar to those in model S, with the MTS concentrated at the buccal root dentin (Figs. 3, 4, 5).

In model A_1_ restored by CR, a higher tensile stress was distributed on the lower defected wall, with values decreasing from the lower dentin-restoration junction to the surrounding areas. The MTS was concentrated at the lower dentin-restoration junction with a value of 43.88 MPa. As the lower restoration border moved apically (models A_2_ and A_3_), locations of concentrated MTS also shifted apically, remaining at the lower dentin-restoration junction. Increased concentrated areas and higher MTS values (47.22 MPa) were observed in model A_2_, while a reduction in concentrated areas and lower MTS values (38.00 MPa) were recorded in model A_3_ compared with model A_1_ (Figs. 3b, 4b, 5b, Table 3).

In model B_1_ restored by CR, the first principal stress distribution pattern as well as the value (93.68 MPa), and the concentrated areas of MTS were similar to those in model B_0_ (93.81 MPa). As the lower restoration border moved apically (models B_2_ and B_3_), locations of concentrated MTS also shifted apically, remaining at the lower dentin-restoration junction. A reduction in concentrated areas and lower values of MTS were observed in models B_2_ and B_3_ (model B_2_: 64.14 MPa, model B_3_: 50.25 MPa) compared with model B_1_ (Figs. 3c, 4c, 5c, Table 3).

In models C_1_, C_2_, and C_3_ restored by CR, the first principal stress distribution patterns were similar to those in model C_0_, with the MTS concentrated at the zeniths. Similar values (model C1: 86.42 MPa, model C_2_: 84.71 MPa) and concentrated areas of MTS were observed in models C_1_ and C_2_, while an increase in concentrated areas and higher value of MTS (166.32 MPa, by two-fold) were found in model C_3_ compared with model C_0_ (86.42 MPa) (Figs. 3d, 4d, 5d, Table 3).

When using MTA, which has a similar elastic modulus to CR, the principal stress distribution patterns, values, and MTS concentrated areas were similar to those restored by CR in all models. When using GIC, which has a lower elastic modulus, the values and concentrated areas of MTS decreased in models A_1_, A_2_, A_3_, B_2_, and B_3_, but were similar to other models, when compared with those restored by CR or MTA. Furthermore, there were no obvious differences in the concentrated locations of MTS nor the principal stress distribution patterns in other regions among models restored by CR, MTA, or GIC (Figs. 3, 4, and 5, Table 3).

**Table 3:** Maximum tensile strength values (MPa)

Model	Enamel			Dentin			Restoration			Total		
	CR	GIC	MTA	CR	GIC	MTA	CR	GIC	MTA	CR	GIC	MTA
S	35.16	35.16	35.16	29.70	29.70	29.70				35.16	35.16	35.16
A_0_	34.26	34.26	34.26	36.40	36.40	36.40				36.40	36.40	36.40
A_1_	34.26	34.26	34.26	31.64	29.53	31.30	34.90	29.16	33.89	43.88	38.54	43.05
A_2_	34.26	34.27	34.26	35.03	32.81	34.49	41.30	33.45	39.87	47.22	40.22	46.09
A_3_	34.27	34.27	34.27	30.35	30.00	30.00	38.00	29.05	36.33	38.00	34.27	36.33
A_4_	34.25	34.26	34.25	31.50	31.52	31.52	15.38	11.70	14.58	34.25	34.26	34.25
B_0_	34.01	34.01	34.01	93.81	93.81	93.81				93.81	93.81	93.81
B_1_	34.01	34.01	34.01	93.68	93.69	93.70	10.63	7.89	10.22	93.68	93.69	93.67
B_2_	34.07	34.07	34.07	43.24	39.28	42.78	44.75	38.00	43.40	64.14	57.26	63.00
B_3_	33.98	33.98	33.98	37.63	33.33	37.10	50.36	41.79	48.71	50.25	43.62	49.11
B_4_	34.17	34.17	34.17	30.69	30.67	30.69	16.96	14.06	16.41	34.17	34.17	34.17
C_0_	34.52	34.52	34.52	86.42	86.42	86.42				86.42	86.42	86.42
C_1_	34.53	34.53	34.53	86.42	86.42	86.42	0.63	0.38	0.60	86.42	86.42	86.42
C_2_	34.52	34.52	34.52	84.71	84.71	84.71	2.94	1.84	2.81	84.71	84.71	84.71
C_3_	34.39	34.39	34.39	166.32	165.56	166.20	35.94	26.91	34.10	166.32	165.56	166.20
C_4_	34.39	34.39	34.39	26.87	26.87	26.87	16.58	14.14	16.25	34.39	34.39	34.39

Total includes all dental tissues and restorations.

*CR* composite resin, *GIC* glass-ionomer cement, *MTA* mineral trioxide aggregate

### Stress distribution at dentin-restoration interfaces

**Fig. 6:**
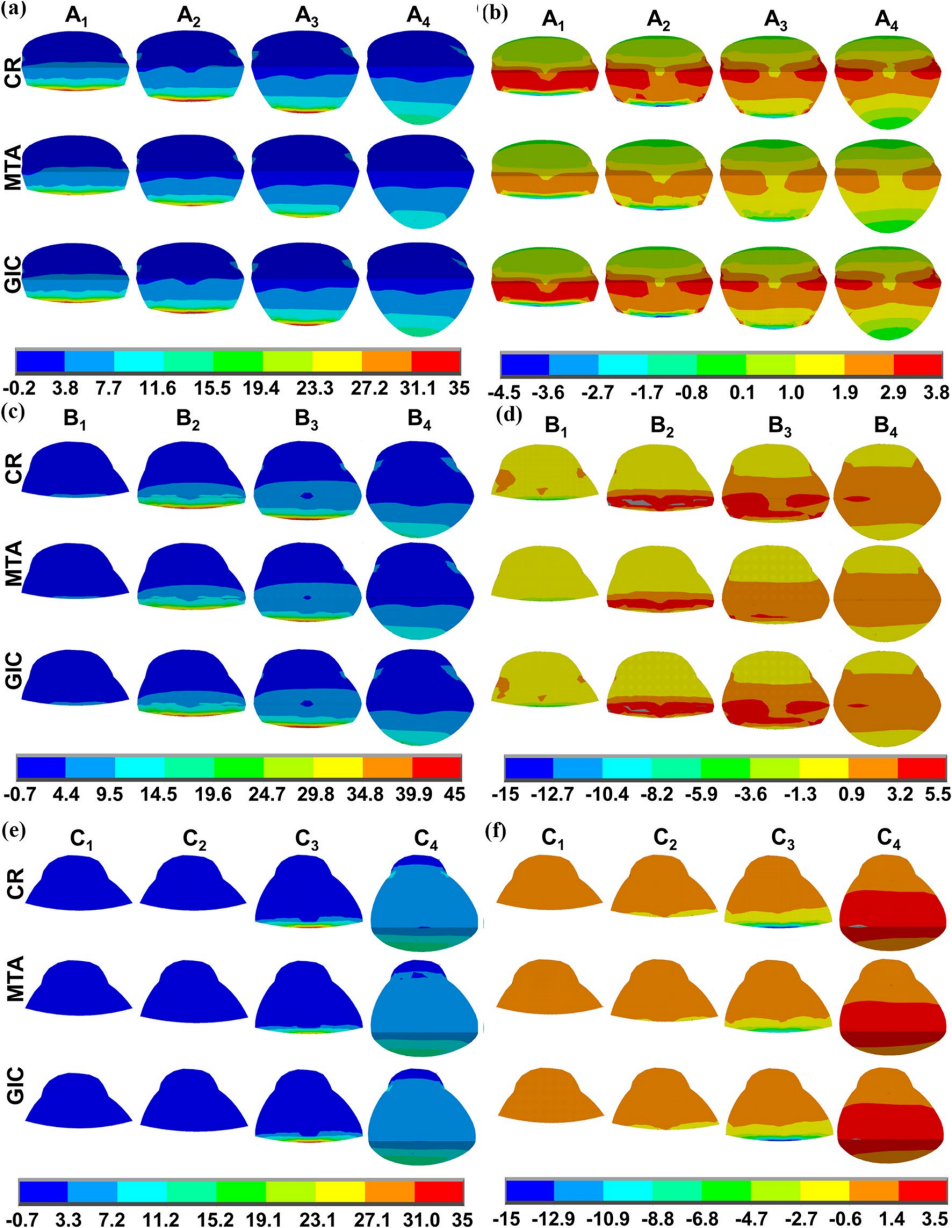
First principal stress and shear stress (MPa) distributions at dentin-restoration interfaces for partial restorations and complete restorations using different restorative materials. **a** First principal stress distributions in partially restored NCCLs (A_1_–A_3_) and completely restored NCCLs (A_4_); **b** shear stress distributions in partially restored NCCLs (A_1_–A_3_) and completely restored NCCLs (A_4_); **c** first principal stress distributions in partially restored NCCLs (B_1_–B_3_) and completely restored NCCLs (B_4_); **d** shear stress distributions in partially restored NCCLs (B_1_–B_3_) and completely restored NCCLs (B_4_); **e** first principal stress distributions in partially restored NCCLs C_1_–C_3_ and completely restored NCCLs (C_4_); **f** shear stress distributions in partially restored NCCLs (C_1_–C_3_) and completely restored NCCLs (C_4_)

In models A_4_, B_4_, and C_4_ restored by CR, tensile stress at the lower interfaces gradually decreased from the lower borders to the zeniths, with the MTS concentrated at the lower border, while low tensile stress was uniformly distributed at the upper interfaces (Figs. 6a, c, e). The shear stress distribution at the interfaces was symmetrical around the zeniths, with the values decreasing from the zeniths to the surrounding regions. The MSS was also concentrated at the zeniths (Fig. 6b, d, f).

In models A_1_, A_2_, A_3_, B_2_, B_3_, and C_3_ restored by CR, the distribution patterns of tensile stress and shear stress at the interfaces were similar to those in complete restorations, but were more uneven. The MTS and MSS were concentrated at lower borders with higher values than those of the corresponding complete restorations. In models B_1_, C_1_, and C_2_ restored by CR, the tensile stress and shear stress were evenly distributed at the interfaces. The MTS and the MSS remained concentrated at the lower border with lower values than those of the corresponding complete restorations (Fig. 6, Table 4).

When using MTA as the restorative material, the stress distribution patterns at the dentin-restoration interfaces, and the MTS and MSS concentrated locations were similar to those using CR; however, the MTS and MSS values were slightly reduced. When GIC was used as the restorative material, the stress distribution trends at the interfaces, and the MTS and MSS concentrated locations were not obviously changed. However, values and concentrated areas of the MTS and MSS were reduced compared to those using CR or MTA (Fig. 6, Table 4).

**Table 4:** Maximum tensile strength and maximum shear strength values (MPa) at dentin-restoration interfaces

Model	CR		GIC		MTA	
	MTS	MSS	MTS	MSS	MTS	MSS
A_1_	34.90	6.21	29.16	5.40	33.89	6.31
A_2_	41.30	7.74	33.45	6.81	39.87	7.37
A_3_	38.00	6.54	29.05	4.66	36.33	6.30
A_4_	15.38	3.73	11.07	2.67	15.58	3.53
B_1_	10.62	5.43	7.89	3.95	10.22	5.20
B_2_	44.75	17.56	38.00	14.39	43.40	17.35
B_3_	50.36	8.69	41.79	7.58	48.71	8.41
B_4_	16.96	3.45	14.06	2.68	16.41	3.38
C_1_	0.63	0.81	0.38	0.54	0.60	0.77
C_2_	2.94	1.50	1.84	0.94	2.81	1.43
C_3_	35.94	16.92	26.91	11.60	34.10	16.02
C_4_	16.58	3.65	14.14	2.98	16.25	3.58
Ultimate tensile strength	92.80		11.80		7.21	
Bond strength	60.00	25.30	9.30	6.30	12.00	2.51

*CR* composite resin, *GIC* glass-ionomer cement, *MTA* mineral trioxide aggregate, *MTS* maximum tensile strength, *MSS* maximum shear strength

According to Table 4, for CR, the MTS and MSS values were lower than the bond strength and UTS in all models. For MTA, the MTS and MSS values were lower than the bond strength and UTS only in models C_1_ and C_2_, while in other models the MTS or MSS exceeded the bond strength or UTS. For GIC, the MTS and MSS values were lower than the bond strength only in models C_1_ and C_2_, while the MTS and MSS exceeded the bond strength in other models, which was similar to MTA. The MTS values of GIC were lower than the UTS in models A_4_, B_1_, C_1_, and C_2_, while the values exceeded the UTS in other models.

## Discussion

Numerous clinical trials have verified the effectiveness of partial restoration combined with periodontal root coverage surgery in the treatment of CDs [8–10]. However, previous FE analyses [20–22] have only focused on the complete restorative treatment of NCCLs, while biomechanical research on partial restorative treatment is lacking. This study was the first to evaluate biomechanical behaviors in partially restored NCCLs according to location of defected zeniths (three levels), location of lower restoration borders (three levels), and restorative material (three types), thereby simulating different clinical situations. The aim was to verify the feasibility of the partial restorative treatment of NCCLs.

There are several reasons for designing the specific NCCL morphology and size selected in this study. The morphology was based on an epidemiological investigation [40], which showed that a NCCL with a horizontal oval surface contour in the coronal plane and wedge-shaped contour in the axial plane was the dominant type associated with stress. The height selected was within the range of measurements from clinical trials [7, 8]. Regarding the depth, restorative treatment is not indicated in a shallow NCCL due to the low fracture resistance of thin restorations [6, 41]. Furthermore, the mean thickness of the buccal radicular dentin is 2.1 mm in extracted human mandibular premolars [42, 43]; therefore, this depth could simulate the maximum depth for conservative restorative treatment of defected zeniths approaching the pulp cavity. The locations of defected zeniths were categorized into three levels with acute angles, which were also within the range of previous FE studies [14]. NCCLs with a wedge-shaped contour [14, 37], deeper depth [14, 21], or more acute angle [44] would increase the magnitude of stress concentration around the zeniths compared to NCCLs with a more rounded contour, shallower depth, or more obtuse angle. Therefore, the specifically designed NCCLs in this study aimed to simulate the most extreme clinical situation.

During the construction of partially restored NCCL models, when complete root coverage is achieved in a non-rotated or non-extruded tooth with Miller class I or II gingival recession after periodontal root coverage surgery, the predetermined MRC line will coincide with the CEJ [45]. Accordingly, the lower restoration border is placed up to 1 mm apically to the MRC (also the CEJ), which explains the reason for constructing models A_1_, B_1_, and C_1_. In other conditions, the MRC line may reside apically to the CEJ [3]; therefore, different partially restored NCCL models were produced.

Consistent with previous studies [20, 21], the present results showed that tensile stress accumulated at defected zeniths in unrestored NCCLs, which would lead to the progression of NCCLs due to the less rigid enamel prisms and dentinal tubules at the cervix. In completely restored NCCLs, the stress distribution patterns were similar to those in the sound tooth. The complete restoration acted as a valid alternative to the lost dental structures, thus dissipating the initial concentrated stress.

In partially restored NCCLs, the stress distribution patterns were mainly influenced by the location of the lower restoration borders relative to the defected zeniths. Specifically, when the zeniths were not covered by the partial restorations, the stress distribution patterns were almost unchanged compared with those in the unrestored NCCLs. This could be attributed to the fact that the lower defected wall dispersed more tensile stress than the upper defected wall under loading. These results also showed that when the partial restoration borders adjoined the defected zeniths, the stress distribution patterns were the worst with significantly increased stress concentrated around the zeniths. This is probably because the joints of dissimilar materials with different elastic moduli would increase the stress concentration around the joints [46, 47]. Sustained concentrated stress can lead to NCCL progression, pulp vitality changes [48], and negative effects on the metabolic activity of human gingival fibroblasts [49]. Therefore, partial restorations of NCCLs that are ineffective at covering the zenith, should be chosen cautiously.

In contrast to the circumstances above, when the zeniths were covered by partial restorations, the initial concentrated stress at the zeniths was obviously alleviated. The MTS was transferred to the lower restoration border, shifting from the central dentin near the pulp cavity to the peripheral dentin. This is because the partial restorations could substitute part of the lost dental structures and strengthen the residual dental structures, further dissipating the initial concentrated stress. It can be speculated that the altered stress distributions will further decrease fracture risks of inner dentin around the zeniths and reduce the progression speed of NCCLs. Therefore, these results indicate that partial restorations covering the defected zenith are effective at improving the stress distributions in NCCLs. Furthermore, they also showed that under this circumstance, the MTS and MSS values in partial restorations would increase and become obviously higher than the values in complete restorations. Since the buffering effect of the CR adhesive layer during the stress transfer process was disregarded, due to its thinness and similar elastic modulus to dentin, the MTS and MSS values transferred to the dentin-restoration interfaces, especially at the lower restoration border, also increased. Thus, partial restorations, particularly at the lower margins, would be likely to undergo biomechanically-related restorative failure, similar to the risk locations in complete restorative treatment [14, 50].

The effects of restorative material type on stress distribution were also investigated in this study. Only when the partial restoration was effective at improving the stress distributions in NCCLs did the elastic modulus of the restorative materials have an obvious effect on the stress distribution. Among the selected restorative materials in the present study, CR and MTA both presented similar elastic moduli to dentin; therefore, the stress distribution in NCCLs restored by CR and MTA were similar. GIC, however, had the lowest elastic modulus and was less rigid. The GIC partial restorations presented the lowest stress values probably because GIC was more prone to deforming and flexing with the dental structures under loading, thus presenting higher strains and accumulating lower stress in the restoration [51]. However, the stress concentrated locations at the interfaces and the stress distribution in other regions were not obviously influenced by the restorative material type, indicating that the overall stress distributions in NCCLs were mainly affected by the restoration location and range.

In the complete restorative treatment of NCCLs, some unfavorable factors of NCCLs, including the lack of retention form, unfavorable lesion marginal locations on the dentin or cementum, and the extensive presence of sclerotic dentin around cervical lesions, will compromise the retention and bonding effectiveness of restorations [39, 52]. These factors should also be considered in partial restorative treatment. Additionally, higher MTS and MSS values in partial restorations compared with complete restorations were observed in the present study, indicating greater debonding risks in partial restorations than complete restorations. A restoration bond failure can be categorized as an adhesive bond failure or cohesive bond failure [53]. Specifically, an adhesive bond failure occurs when the MTS or MSS values at the interfaces exceed the bond strength of the self-adhesive restorative materials or adhesives to dentin, while a cohesive bond failure occurs when the MTS at the interfaces exceeds the UTS of the restorative materials or adhesives. According to the abovementioned results, CR had the highest elastic modulus among the three restorative materials and was less likely to deform under the same loading, thus absorbing more stress in the restoration. Although the highest stress values and concentrated areas were transferred to the interfaces, the MTS and MSS values at the interfaces were still lower than the bond strength of the adhesives and UTS of the adhesives or CR, indicating no debonding risk. This is attributed to the excellent mechanical properties of CR and strong bond strength of current dentin adhesives. Contrasting results were obtained for GIC and MTA. GIC had the lowest elastic modulus among the selected materials, transferring the lowest stress values with the smallest concentration areas to the interfaces. However, due to the suboptimal mechanical properties and poor bond strength of GIC, the MTS and MSS values exceeded the bond strength and UTS of GIC under most conditions, except in models C_1_ and C_2_, indicating obviously greater debonding risks. MTA had a similar elastic modulus and presented a similar stress distribution pattern to CR, but had a lower bond strength and less favorable mechanical properties. Therefore, the MTS and MSS values far exceeded the bond strength and UTS of MTA under most circumstances, except in models C_1_ and C_2_, similarly indicating high debonding risks. In addition, some other physical properties such as compressive strength, flexural strength, and microhardness should also be taken into consideration when choosing a suitable restorative material. The physical properties of MTA were obviously inferior to those of GIC and CR, while CR presented the best physical properties among these three materials [24, 54–58]. Taken together, these findings suggest that CR is preferential for restoring NCCLs, especially in partial restorative treatment, and can decrease potential restoration debonding failures and prolong the longevity of restorations.

It should be emphasized that it was not possible to directly extrapolate actual clinical results in the present study and the findings should therefore be interpreted cautiously. This study has a few other limitations. Firstly, the bond strength of different materials can be measured by a variety of methods without standardized laboratory protocols. The present study only adopted two of the most commonly used parameters, the macro shear bond strength and the micro-tensile bond strength, as the references. Furthermore bond strength measurements are affected by several complex factors including substrate [59–63], specimen [64–66], and test mechanic-related factors [67–69]; therefore, their ranges vary among studies. Secondly, cervical restoration retention can be affected by many factors such as tooth-related factors (presence of hyper-mineralized sclerotic dentin, histological and morphological conditions of enamel prisms, and dentinal tubules at the cervix), environment-related factors (dynamic loading, thermal cycles, moisture environment, and microorganisms associated with an acidic environment), patient-related factors (age, parafunctional habits, and oral hygiene conditions), operator-related factors (cavity preparation methods and isolation), and other material-related factors (polymerization shrinkage of resinous materials, type of adhesive product, adhesive layer thickness, and chemical degradation) [39, 70]. Considering these various factors, cervical restoration debonding failure is more likely to occur in the oral cavity than in the constructed models. Thirdly, when constructing the FE models, they were simplified in order to highlight the main experimental factors, while other delicate clinical operations such as enamel bevel construction at the coronal NCCL borders [7], odontoplasty of the apical NCCL borders [8], and rounding the internal angles of the defected zeniths [14] were not simulated. Therefore, FE analyses based on more complex models, as well as associated clinical trials with long observation periods, are needed in the future.

## Conclusion

Within the limitations of this study, the following conclusions can be made:

1. Partial restorations that fully cover the defected zeniths can improve the stress distributions around the defected zenith in NCCLs; however, they are compromised compared with complete restorations.
2. Partial restorations that are unable to cover the defected zeniths have no effect on stress distributions or can even worsen the situation.
3. Composite resin with currently used dentin adhesives presented the lowest risk of bonding failure, and should therefore be the first choice in partial restorative treatment.

## Data Availability

The datasets used or analyzed during the current study are available from the corresponding author on reasonable request.

## Abbreviations

NCCL: Non-carious cervical lesion
CR: Composite resin
GIC: Glass-ionomer cement
MTA: Mineral trioxide aggregate
CEJ: Cementoenamel junction
CD: Combined defect;
MRC: Maximum root coverage level
FE: Finite element
MTS: Maximum tensile stress;
MSS: Maximum shear stress
UTS: Ultimate tensile strength

## References

[1] Teixeira DNR, Thomas RZ, Soares PV, Cune MS, Gresnigt MMM, Slot DE (2020). Prevalence of noncarious cervical lesions among adults: a systematic review. J Dent.

[2] Pini-Prato G, Franceschi D, Cairo F, Nieri M, Rotundo R (2010). Classification of dental surface defects in areas of gingival recession. J Periodontol.

[3] Zucchelli G, Testori T, De Sanctis M (2006). Clinical and anatomical factors limiting treatment outcomes of gingival recession: a new method to predetermine the line of root coverage. J Periodontol.

[4] Zucchelli G, Mounssif I (2015). Periodontal plastic surgery. Periodontol 2000.

[5] Zucchelli G, Gori G, Mele M, Stefanini M, Mazzotti C, Marzadori M, Montebugnoli L, De Sanctis M (2011). Non-carious cervical lesions associated with gingival recessions: a decision-making process. J Periodontol.

[6] Santamaria MP, Mathias-Santamaria IF, Ferraz LFF, Casarin RCV, Romito GA, Sallum EA, Pini-Prato GP, Casati MZ (2021). Rethinking the decision-making process to treat gingival recession associated with non-carious cervical lesions. Braz Oral Res.

[7] Mathias-Santamaria IF, Silveira CA, Rossato A, Sampaio de Melo MA, Bresciani E, Santamaria MP (2021). Single gingival recession associated with non-carious cervical lesion treated by partial restoration and coronally advanced flap with or without xenogenous collagen matrix: a randomized clinical trial evaluating the coverage procedures and restorative protocol. J Periodontol.

[8] Santamaria MP, Silveira CA, Mathias IF, Neves F, Dos Santos LM, Jardini MAN, Tatakis DN, Sallum EA, Bresciani E (2018). Treatment of single maxillary gingival recession associated with non-carious cervical lesion: randomized clinical trial comparing connective tissue graft alone to graft plus partial restoration. J Clin Periodontol.

[9] Santamaria MP, Suaid FF, Casati MZ, Nociti FH, Sallum AW, Sallum EA (2008). Coronally positioned flap plus resin-modified glass ionomer restoration for the treatment of gingival recession associated with non-carious cervical lesions: a randomized controlled clinical trial. J Periodontol.

[10] Cairo F, Cortellini P, Nieri M, Pilloni A, Barbato L, Pagavino G, Tonetti M (2020). Coronally advanced flap and composite restoration of the enamel with or without connective tissue graft for the treatment of single maxillary gingival recession with non-carious cervical lesion. A randomized controlled clinical trial. J Clin Periodontol.

[11] Aslan T, Üstün Y, Esim E (2019). Stress distributions in internal resorption cavities restored with different materials at different root levels: a finite element analysis study. Aust Endod J.

[12] Aslan T, Esim E, Üstün Y, Dönmez Özkan H (2021). Evaluation of stress distributions in mandibular molar teeth with different iatrogenic root perforations repaired with biodentine or mineral trioxide aggregate: a finite element analysis study. J Endod.

[13] Schwartz-Dabney CL, Dechow PC (2003). Variations in cortical material properties throughout the human dentate mandible. Am J Phys Anthropol.

[14] Ichim I, Schmidlin PR, Kieser JA, Swain MV (2007). Mechanical evaluation of cervical glass-ionomer restorations: 3D finite element study. J Dent.

[15] Silveira CA, Mathias IF, da Silva Neves FL, Castro Dos Santos NC, Araujo CF, Neves Jardini MA, Bresciani E, Santamaria MP (2017). Connective tissue graft and crown-resin composite restoration for the treatment of gingival recession associated with noncarious cervical lesions: case series. Int J Periodontics Restor Dent.

[16] Cairo F, Nieri M, Pagliaro U (2014). Efficacy of periodontal plastic surgery procedures in the treatment of localized facial gingival recessions. A systematic review. J Clin Periodontol.

[17] Roccuzzo M, Bunino M, Needleman I, Sanz M (2002). Periodontal plastic surgery for treatment of localized gingival recessions: a systematic review. J Clin Periodontol.

[18] de Menezes FCH, da Silva SB, Valentino TA, Oliveira MAHM, Rastelli ANS, Conçalves LS (2013). Evaluation of bond strength and thickness of adhesive layer according to the techniques of applying adhesives in composite resin restorations. Quintessence Int.

[19] Richert R, Farges JC, Tamimi F, Naouar N, Boisse P, Ducret M (2020). Validated finite element models of premolars: a scoping review. Materials (Basel).

[20] Soares PV, Machado AC, Zeola LF, Souza PG, Galvao AM, Montes TC, Pereira AG, Reis BR, Coleman TA, Grippo JO (2015). Loading and composite restoration assessment of various non-carious cervical lesions morphologies—3D finite element analysis. Aust Dent J.

[21] Zeola LF, Pereira FA, Machado AC, Reis BR, Kaidonis J, Xie Z, Townsend GC, Ranjitkar S, Soares PV (2016). Effects of non-carious cervical lesion size, occlusal loading and restoration on biomechanical behaviour of premolar teeth. Aust Dent J.

[22] Machado AC, Soares CJ, Reis BR, Bicalho AA, Raposo L, Soares PV (2017). Stress-strain analysis of premolars with non-carious cervical lesions: influence of restorative material, loading direction and mechanical fatigue. Oper Dent.

[23] Soares PV, Santos-Filho PCF, Queiroz EC, Araujo TC, Campos RE, Araujo CA, Soares CJ (2008). Fracture resistance and stress distribution in endodontically treated maxillary premolars restored with composite resin. J Prosthodont.

[24] Della Bona A, Benetti P, Borba M, Cecchetti D (2008). Flexural and diametral tensile strength of composite resins. Braz Oral Res.

[25] Pai S, Naik N, Patil V, Kaur J, Awasti S, Nayak N (2019). Evaluation and comparison of stress distribution in restored cervical lesions of mandibular premolars: three-dimensional finite element analysis. J Int Soc Prev Community Dent.

[26] Jordehi AY, Ghasemi A, Zadeh MM, Fekrazad R (2007). Evaluation of microtensile bond strength of glass ionomer cements to dentin after conditioning with the Er, Cr:YSGG Laser. Photomed Laser Surg.

[27] Moshaverinia A, Roohpour N, Billington RW, Darr JA, Rehman IU (2008). Synthesis of N-vinylpyrrolidone modified acrylic acid copolymer in supercritical fluids and its application in dental glass-ionomer cements. J Mater Sci Mater Med.

[28] Moshaverinia A, Roohpour N, Ansari S, Moshaverinia M, Schricker S, Darr JA, Rehman IU (2009). Effects of N-vinylpyrrolidone (NVP) containing polyelectrolytes on surface properties of conventional glass-ionomer cements (GIC). Dent Mater.

[29] Brito-Junior M, Pereira RD, Verissimo C, Soares CJ, Faria-e-Silva AL, Camilo CC, Sousa-Neto MD (2014). Fracture resistance and stress distribution of simulated immature teeth after apexification with mineral trioxide aggregate. Int Endod J.

[30] Demirel A, Bezgin T, Sari S (2021). Effects of root maturation and thickness variation in coronal mineral trioxide aggregate plugs under traumatic load on stress distribution in regenerative endodontic procedures: a 3-dimensional finite element analysis study. J Endod.

[31] Frankenberger R, Nassiri S, Lucker S, Lygidakis NN, Kramer N (2021). The effect of different liners on the bond strength of a compomer to primary teeth dentine: in vitro study. Eur Arch Paediatr Dent.

[32] Garcia L, Rossetto HL, Pires-de-Souza FCP (2014). Shear bond strength of novel calcium aluminate-based cement (EndoBinder) to root dentine. Eur J Dent.

[33] Dianat O, Naseri M, Tabatabaei SF (2017). Evaluation of properties of mineral trioxide aggregate with methyl cellulose as liquid. J Dent (Tehran).

[34] Abdalla AI (2004). Microtensile and tensile bond strength of single-bottle adhesives: a new test method. J Oral Rehabil.

[35] Harnirattisai C, Roengrungreang P, Rangsisiripaiboon U, Senawongse P (2012). Shear and micro-shear bond strengths of four self-etching adhesives measured immediately and 24 hours after application. Dent Mater J.

[36] Ikeda T, De Munck J, Shirai K, Hikita K, Inoue S, Sano H, Lambrechts P, Van Meerbeek B (2005). Effect of evaporation of primer components on ultimate tensile strengths of primer-adhesive mixture. Dent Mater.

[37] Guimarães JC, Guimarães Soella G, Brandão Durand L, Horn F, Narciso Baratieri L, Monteiro S, Belli R (2014). Stress amplifications in dental non-carious cervical lesions. J Biomech.

[38] Giannini M, Soares CJ, de Carvalho RM (2004). Ultimate tensile strength of tooth structures. Dent Mater.

[39] Santos MJ, Ari N, Steele S, Costella J, Banting D (2014). Retention of tooth-colored restorations in non-carious cervical lesions–a systematic review. Clin Oral Investig.

[40] Igarashi Y, Yoshida S, Kanazawa E (2017). The prevalence and morphological types of non-carious cervical lesions (NCCL) in a contemporary sample of people. Odontology.

[41] Rocca GT, Baldrich B, Saratti CM, Delgado LM, Roig M, Daher R, Krejci I (2021). Restoration's thickness and bonding tooth substrate are determining factors in minimally invasive adhesive dentistry. J Prosthodont Res.

[42] Bellucci C, Perrini N (2002). A study on the thickness of radicular dentine and cementum in anterior and premolar teeth. Int Endod J.

[43] Pilloud MA, Hillson S (2012). Brief communication: the use of alternative dental measurements on deciduous teeth. Am J Phys Anthropol.

[44] Soares PV, Santos-Filho PCF, Soares CJ, Faria VLG, Naves MF, Michael JA, Kaidonis JA, Ranjitkar S, Townsend GC (2013). Non-carious cervical lesions: influence of morphology and load type on biomechanical behaviour of maxillary incisors. Aust Dent J.

[45] Miller PD (1985). A classification of marginal tissue recession. Int J Periodontics Restor Dent.

[46] Arezoodar AF, Baladi A (2011). The effects of materials properties & angle junction on stress concentration at interface of dissimilar materials. Adv Mater Res.

[47] Bogy DB (1971). Two edge-bonded elastic wedges of different materials and wedge angles under surface tractions. Trans ASME J Appl Mech.

[48] Mjör IA (2001). Pulp-dentin biology in restorative dentistry. Part 5: clinical management and tissue changes associated with wear and trauma. Quintessence Int.

[49] Grunheid T, Zentner A (2005). Extracellular matrix synthesis, proliferation and death in mechanically stimulated human gingival fibroblasts in vitro. Clin Oral Investig.

[50] Ichim IP, Schmidlin PR, Li Q, Kieser JA, Swain MV (2007). Restoration of non-carious cervical lesions Part II. Restorative material selection to minimise fracture. Dent Mater.

[51] Heymann HO, Sturdevant JR, Bayne S, Wilder AD, Sluder TB, Brunson WD (1991). Examining tooth flexure effects on cervical restorations: a two-year clinical study. J Am Dent Assoc.

[52] Kim JH, Cho J, Lee Y, Cho BH (2017). The survival of class V composite restorations and analysis of marginal discoloration. Oper Dent.

[53] Ebnesajjad S. Introduction and adhesion theories. 2011:3–13.

[54] Nica I, Iovan G, Stoleriu S, Ghiorghe CA, Andrian S (2018). Comparative study regarding the compressive strength of different composite resins used for direct restorations. Mater Plast.

[55] Fuhrmann D, Murchison D, Whipple S, Vandewalle K (2020). Properties of new glass-ionomer restorative systems marketed for stress-bearing areas. Oper Dent.

[56] Bao X, Liu F, He J (2021). Preparation of basalt fibers grafted with amine terminated urea-based oligomer and its application in reinforcing conventional glass ionomer cement. J Mech Behav Biomed Mater.

[57] Walker MP, Diliberto A, Lee C (2006). Effect of setting conditions on mineral trioxide aggregate flexural strength. J Endod.

[58] Natu VP, Dubey N, Loke GC, Tan TS, Ng WH, Yong CW, Cao T, Rosa V (2015). Bioactivity, physical and chemical properties of MTA mixed with propylene glycol. J Appl Oral Sci.

[59] Nakamichi I, Iwaku M, Fusayama T (1983). Bovine teeth as possible substitutes in the adhesion test. J Dent Res.

[60] Oztürk B, Malkoç S, Koyutürk AE, Catalbas B, Ozer F (2008). Influence of different tooth types on the bond strength of two orthodontic adhesive systems. Eur J Orthod.

[61] Hevinga MA, Opdam NJ, Frencken JE, Truin GJ, Huysmans MCDNJM (2010). Does incomplete caries removal reduce strength of restored teeth?. J Dent Res.

[62] Pashley EL, Tao L, Matthews WG, Pashley DH (1993). Bond strengths to superficial, intermediate and deep dentin in vivo with four dentin bonding systems. Dent Mater.

[63] Yuan Y, Shimada Y, Ichinose S, Sadr A, Tagami J (2007). Effects of dentin characteristics on interfacial nanoleakage. J Dent Res.

[64] Shono Y, Terashita M, Pashley EL, Brewer PD, Pashley DH (1997). Effects of cross-sectional area on resin-enamel tensile bond strength. Dent Mater.

[65] dos Santos PH, Sinhoreti MAC, Consani S, Sobrinho LC, Adabo GL, Vaz LG (2005). Effect of cyclic compressive loading on the bond strength of an adhesive system to dentin after collagen removal. J Adhes Dent.

[66] Gomes GM, Gomes OMM, Reis A, Gomes JC, Loguercio AD, Calixto AL (2013). Effect of operator experience on the outcome of fiber post cementation with different resin cements. Oper Dent.

[67] Hara AT, Pimenta LA, Rodrigues AL (2001). Influence of cross-head speed on resin-dentin shear bond strength. Dent Mater.

[68] Dao Luong MN, Shimada Y, Turkistani A, Tagami J, Sumi Y, Sadr A (2016). Fractography of interface after microtensile bond strength test using swept-source optical coherence tomography. Dent Mater.

[69] Montagner AF, Opdam NJ, Ruben JL, Cenci MS, Huysmans M-C (2016). Bonding effectiveness of composite-dentin interfaces after mechanical loading with a new device (Rub&Roll). Dent Mater J.

[70] Stewardson DA, Thornley P, Bigg T, Bromage C, Browne A, Cottam D, Dalby D, Gilmour J, Horton J, Roberts E (2011). The survival of Class V restorations in general dental practice. Part 2, early failure. Br Dent J.

